# Synergistic Effects of Lauric Arginate and Peracetic Acid in Reducing *Listeria monocytogenes* on Fresh Apples

**DOI:** 10.3389/fmicb.2021.641034

**Published:** 2021-06-18

**Authors:** Xiaoye Shen, Jian Cong, Joshua Mugendi, Ines Hanrahan, Mei-Jun Zhu

**Affiliations:** ^1^School of Food Science, Washington State University, Pullman, WA, United States; ^2^Washington Tree Fruit Research Commission, Wenatchee, WA, United States

**Keywords:** *Listeria monocytogenes*, peracetic acid, lauric arginate, apples, spray application, temperature

## Abstract

Apples are naturally coated with a water-repelling hydrophobic wax layer, which may limit the antimicrobial efficacies of surface sanitizer solutions. Lauric arginate (LAE) is a cationic surfactant with antimicrobial efficacy against *Listeria monocytogenes*. In this study, we investigated the antimicrobial and the wettability effects of LAE in enhancing anti-*L. monocytogenes* efficacy of peracetic acid (PAA) and further verified the optimized treatment combinations in a pilot spray-bar brush bed system. Apples after 48 h of inoculation were treated with PAA surface sanitation in combination with different concentrations of LAE at 22 or 46°C. The effectiveness of PAA with LAE solutions in decontaminating *L. monocytogenes* significantly increased with the increased concentration of PAA (60–80 ppm) or LAE (0.01–0.05%) or the treatment temperature (from 22 to 46°C). A 30–120-sec wash by 80 ppm PAA with 0.01 and 0.05% LAE at 22°C reduced *L. monocytogenes* on apples by 2.10–2.25 and 2.48–2.58 log_10_ CFU/apple, respectively. Including LAE in the PAA solution decreased contact angles on apple surfaces. However, the increased wettability of the sanitizer solution may not be the main contributor to the enhanced antimicrobial efficacy of the PAA solution, given that the addition of Tween 80 or Tween 85 only slightly boosted the anti-*L. monocytogenes* efficacy of PAA solutions though both increased the wettability of the PAA solutions. The synergistic effects of PAA and LAE were further validated in a pilot spray-bar brush bed packing system, where a 30-sec spray wash with 80 ppm PAA and 0.05% LAE at 22 and 46°C caused 1.68 and 2.08 log reduction of *Listeria* on fresh apples, respectively. This study provides an improved PAA process/preventive strategy for ensuring microbial food safety of fresh apples that is applicable to commercial apple packing lines.

## Introduction

*Listeria monocytogenes* can potentially transfer to fresh produce including apples during the postharvest handling, which results in deadly outbreaks (McCollum et al., 2013; Angelo et al., 2017). Although listeriosis is rare, the mortality rate of listeriosis is very high (∼ 21%) (Silk et al., 2013). The recent foodborne outbreak of *L. monocytogenes* linked to caramel apples (Angelo et al., 2017) and multiple recall linked to fresh apples due to the potential contamination of *L. monocytogenes* (FDA, 2015, 2017b) highlight the importance of effective control strategies to minimize contamination risk of *L. monocytogenes* on fresh apples.

Antimicrobial wash interventions including chlorine-based sanitizers (Du et al., 2002; Rodgers et al., 2004; Sheng et al., 2020a), organic acids such as lactic acid and citric acid (Park et al., 2011), and peracetic acid (PAA) (Rodgers et al., 2004; Shen et al., 2019) were used to decontaminate *L. monocytogenes* on fresh apples. Of these antimicrobials, PAA is the most widely used sanitizer in apple packing lines during the spray-bar brush bed intervention (Zhu et al., 2020). PAA has broad-spectrum antimicrobial activity (Baert et al., 2009) and does not produce toxic by-products (Monarca et al., 2002). PAA applied at 80 ppm, a concentration approved by the Food and Drug Administration (FDA) to wash fresh produce without further rinsing requirement (FDA, 2017a), is more efficient in decontaminating *L. monocytogenes* on fresh apples compared with 100 ppm chlorine-based sanitizers (Shen et al., 2019; Sheng et al., 2020a). However, spray wash of 80 ppm PAA at the current industry practice (at ambient temperature for 30–120 sec contact time) only resulted in about one log reduction of *L. monocytogenes* (Shen et al., 2020), indicating the need to boost the anti-*Listeria* efficacy of PAA.

Fresh apples are naturally covered with a hydrophobic wax layer (Dong et al., 2012; Veraverbeke et al., 2001), which might limit the direct contact of sanitizer solutions on apple surfaces. Surfactants are amphipathic molecules that exist in hydrophobic/hydrophilic interfaces resulting in easy spreading of a liquid solution (Nakama, 2017). Lauric arginate (LAE) is a cationic surfactant derived from lauric acid, L-arginine, and ethanol (Infante et al., 1984), which was approved by the FDA as Generally Recognized as Safe (GRAS) when used at the concentration of 0.02% for direct addition to food products such as meat, cheese, and fruit juice (FDA, 2005). LAE has antimicrobial activity against *Listeria* when used alone or in combination with other sanitizers. Application of 0.01% LAE for 120 sec caused 3.1 log reduction of *L. monocytogenes* on lettuces at an initial bacterial population of 6.6 log_10_ CFU/g (Nübling et al., 2017). A 20-min treatment of 0.10% LAE reduced *Listeria innocua* by 2.3 log_10_ CFU/cm^2^ on lettuce surfaces (Huang and Nitin, 2017). A 3-min of 0.02% LAE exposure resulted in 1 log_10_ CFU/cm^2^ reduction of *L. monocytogenes* biofilm on lettuce surfaces (Sadekuzzaman et al., 2017). LAE was reported to enhance the anti-*Listeria* efficacy of potassium lactate and sodium diacetate on dairy and meat products (Stopforth et al., 2010; Soni et al., 2012). Existing data collectively suggest that LAE is a promising antimicrobial substance for the control of *L. monocytogenes* on food products. However, a recent laboratory scale study reported that the antimicrobial efficacy of 80 ppm PAA with 0.10% LAE against *L. innocua* on apple surfaces was not significantly different from that of 80 ppm PAA alone (Pietrysiak et al., 2019). Currently, no information is available about the strengthening effects of LAE in PAA solutions against *L. monocytogenes* on fresh apples.

The objectives of this study were to evaluate and optimize the effects of LAE in strengthening the anti-*Listeria* efficacy of PAA on fresh apples and further verify the optimized PAA and LAE treatments in a pilot spray-bar brush bed apple processing system. This study provides a practical and optimized PAA intervention strategy for the apple industry and other produce industries with similar postharvest handling and processing for their food safety programs.

## Materials and Methods

### Bacterial Inoculum Preparation

Three *L. monocytogenes* strains, NRRL B-57618 (1/2a, 2011 cantaloupe outbreak isolate), NRRL-33466 (1/2b, processing plant isolate), and NRRL B-33053 (4b, 1983 coleslaw outbreak isolate) and *L. innocua* strains (NRRL B-33197, NRRL B-33314, and NRRL B-33554) were obtained from USDA-ARS culture collection [National Center for Agricultural Utilization Research (NRRL), Peoria, IL, United States). Stock cultures were stored at −80°C in trypticase soy broth [Becton, Dickinson and Company (BD), Sparks, MD, United States) supplemented with 6 g/L yeast extract (Fisher Scientific, Fair Lawn, NJ, United States) (TSBYE) and 20% (*v*/*v*) glycerol (J. T. Baker, Philipsburg, NJ, United States). Each frozen stock culture was subcultured in TSBYE at 37°C for 24 h and transferred into fresh TSBYE for a second 24-h subculture. Following incubation, the cultures were centrifuged at 8,000 × *g* for 5 min at 4°C and the resulting pellets were washed once and resuspended in sterile phosphate-buffered saline (PBS, pH 7.4) (EMD Millipore, Billerica, MA, United States). For apple inoculation, a three-strain *L. monocytogenes* or *L. innocua* cocktail was used, and each strain suspension was mixed in equal proportions and diluted to achieve ∼ 10^6^ CFU/ml in sterile PBS solutions.

### Preparation of Sanitizer Solutions

Cationic surfactant LAE (CytoGuard^TM^) was kindly provided by A&B Ingredients, Inc. (Field, NJ, United States). Nonionic surfactants of Tween 80 and Tween 85 were purchased from Sigma (St. Louis, MO, United States). Bioside HS (a stabilized mixture of 15% PAA and 22% hydrogen peroxide) (Pace International Inc., Wapato, WA, United States) was used to prepare the 60–80 ppm PAA solutions. The concentration of PAA in the respective solution was verified using a titration kit (Aquaphoenix Scientific, Hanover, PA, United States). LAE (0.001–0.10%, *v*/*v*), Tween 80 (0.10–0.20%, *v*/*v*) or Tween 85 (0.10–0.20%, *v*/*v*) were added to 80 ppm PAA solution to the specified concentration.

### The Intervention of LAE With PAA Solutions Against *L. monocytogenes* in Water

The above prepared PAA with LAE solutions were individually inoculated with three-strain *L. monocytogenes* cocktail at 5 × 10^8^ CFU/ml, where 1 ml of three-strain *L. monocytogenes* cocktail suspension was added to 9 ml of respective PAA with LAE solution, treated for 30 sec, then the 1.0 ml of solution was sampled and immediately neutralized with 9.0 ml of D/E neutralizing broth (BD). The survivals were enumerated by 10-fold serial dilution with sterile PBS and plating on duplicate TSAYE (TSBYE with 1.5% agar) plates and incubated at 37°C for 24 h. The detection limit of *L. monocytogenes* is 10 CFU/ml.

### Apple Inoculation

Medium-sized (210–230 g) unwaxed Granny Smith apples (GSA) and Fuji apples devoid of cuts, bruising, or scars were used for this study. Before inoculation, 30 apples were randomly sampled for background microflora enumeration, which is about 3.3–4.0 log_10_ CFU/apples. To inoculate, apples were washed with cold tap water, equilibrated to room temperature (∼22°C, RT) overnight, then dip inoculated with the three-strain *L. monocytogenes* or *L. innocua* cocktail inoculum (∼ 6.8 Log_10_ CFU/ml) solution to have ∼ 6 log_10_ CFU/apple inoculation level (Sheng et al., 2017). The inoculated apples were held at RT for 48 h when apples were treated with different sanitizer solutions. For each batch of the inoculated apples, 10 inoculated apples were randomly sampled 0 and 48 h after inoculation to evaluate the initial *L. monocytogenes* or *L. innocua* population and the uniformity on apples.

### Sanitizing Treatment of Apples

The inoculated apples after 48 h inoculation were subjected to 80 ppm PAA solutions in the presence of 0.001–0.10% LAE at RT for a 2-min exposure unless specified. PAA with 0.10–0.20% Tween 80 or Tween 85 were included as a control.

We previously reported that PAA solution when applied at 46°C had enhanced antimicrobial activity against *L. monocytogenes* on apples compared with that at 22°C without compromising the quality of fruit (Shen et al., 2019). Therefore, the antimicrobial efficacies of PAA in combination with 0.01 or 0.05% LAE solutions were further evaluated at 46°C. Each experiment was independently repeated three times; 10 apples per treatment within an independent study. There were 30 apples in total for a selected treatment.

### Evaluation of Antimicrobial Efficacy in a Pilot Scale Spray-Bar System

The spray wash of apples was conducted in a pilot spray-bar processing system equipped with a brush bed at Washington State University. *L. innocua*, a well-defined surrogate of *L. monocytogenes* (Sheng et al., 2020b; Zhu et al., 2020), was used in the pilot spray bar intervention. GSA inoculated with *L. innocua* were spray washed by 80 ppm PAA with or without LAE at 0.01 and 0.05% (*v*/*v*) at the specific temperature (22 or 46°C) for 30 or 120 sec. The flow rate of the spray bar was 0.977 L/min, and the brush bed is rotating at 47 revolutions per min. Each experiment was independently repeated three times with 10 inoculated apples per treatment within each independent study.

### Microbiological Analysis

Immediately after sanitizer interventions, each apple was transferred into a stomacher bag with the addition of 10 ml of D/E neutralizing broth (BD). Each apple was hand-rubbed for 90 s as previously reported (Shen et al., 2019). Rub solutions were 10-fold serially diluted with sterile PBS, and the appropriate dilutions were plated in duplicate on TSAYE plates. The above TSAYE plates were first incubated at 35 ± 2°C for 4 h and then overlaid with a thin layer of modified Oxford agar (MOX, BD) to facilitate the recovery of the injured *Listeria* and to discern *Listeria* from apple background microflora (Kang and Fung, 1999; Shen et al., 2019).

### Contact Angle Measurement

The contact angles of each wash solution on apple surfaces were measured using the Theta Lite Tensiometer (Biolin Scientific, Stockholm, Sweden) at RT. Apple disks with a 2-cm diameter were cut from fresh apples using a sharp knife. To measure the contact angle, 10 μl of wash solution was deposited onto the above-prepared apple disk using a microliter syringe needle, then the static contact angle was recorded within 5-sec contact. To accurately measure the wettability, the contact angle of each solution was measured on 20 apple disks. Mean values of 20 replicates were reported. The larger water contact angle represents the lower wettability.

### Statistical Analysis

Mean differences were compared by one-way analysis of variance (ANOVA) and analyzed by Tukey’s multiple-comparison test (*P* < 0.05) using IBM SPSS 19.0 (Chicago, IL, United States). Microbiological data were reported as mean ± SEM (standard error mean) averaged from three independent experiments with 10 apples per treatment in each independent study, *n* = 30. Contact angles were presented as mean ± SEM, calculated from 20 apple disks, *n* = 20.

## Results

### Efficacies of Peracetic Acid With Lauric Arginate Against *L. monocytogenes* in Water

**FIGURE 1:**
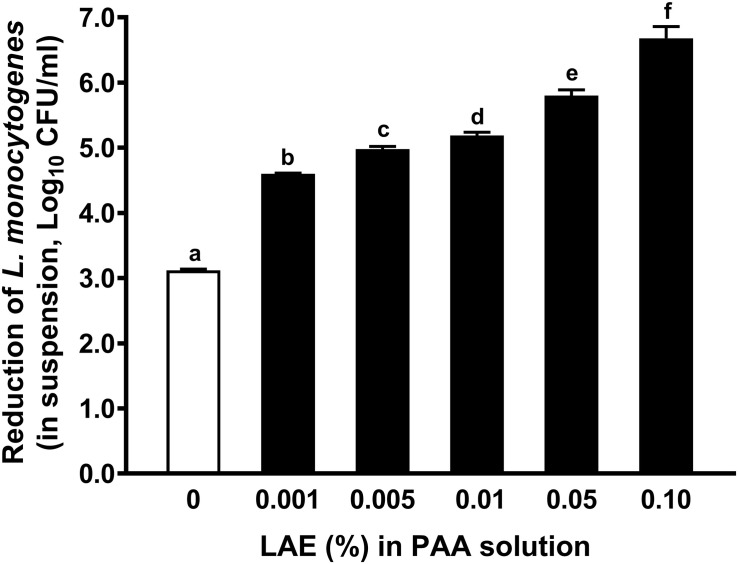
Antimicrobial efficacy of 80 ppm peracetic acid with different concentrations of lauric arginate against *L. monocytogenes* in water. The treatment time is 30 sec. The initial *L. monocytogenes* population was ∼ 7.06 log_10_ CFU/ml. In all treatments, peracetic acid (PAA) was used at 80 ppm. Data are reported as mean ± SEM from three independent studies; there are three replicates per treatment within an independent study. ^a–f^Mean among bars without common letters differ significantly (*P <* 0.05). LAE, lauric arginate.

Adding LAE as low as 0.001% significantly (*P* < 0.05) enhanced anti-*Listeria* efficacy of 80 ppm PAA, which resulted in an additional ∼ 1.48 log_10_ CFU/ml reduction of *L. monocytogenes* in water compared with that treated with 80 ppm PAA alone (caused 3.12 ± 0.02 log_10_ CFU/ml) (Figure 1). The addition of 0.01% LAE in 80 ppm PAA solution caused more than a 5-log reduction of *L. monocytogenes* in water (Figure 1).

### Antimicrobial Efficacy and Wettability of Peracetic Acid Solutions With or Without Surfactant Against *L. monocytogenes* on Fresh Apples

**FIGURE 2:**
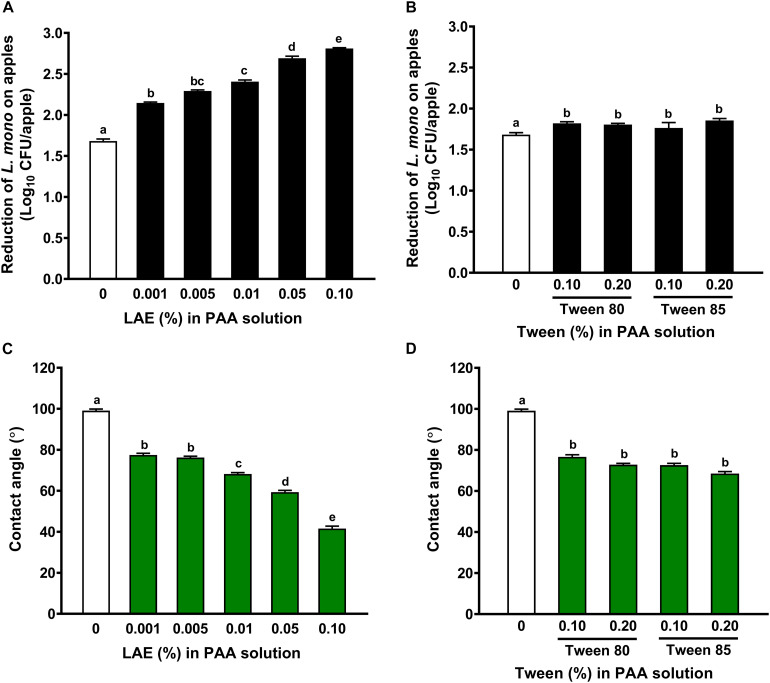
The effects of lauric arginate in enhancing the efficacies of peracetic acid against *L. monocytogenes* on Granny Smith apples may not be due to its wettability. Reduction of *L. monocytogenes* on apples treated with 80 ppm PAA in the presence of lauric arginate (LAE) **(A)** or Tween 80 or Tween 85 **(B)** for 2 min. The wettability of 80 ppm PAA solution with LAE **(C)** or Tween 80 or Tween 85 **(D)** as indicated by their contact angle on apple surfaces. Mean ± SEM, *n* = 30 for the reduction of *L. monocytogenes* and 20 for the contact angle. ^a–e^Mean among bars without common letters differ significantly (*P <* 0.05).

The addition of LAE as low as 0.001% enhanced (*P* < 0.05) the effectiveness of PAA against *L. monocytogenes* on fresh apples (Figure 2A). A 2-min exposure of 80 ppm PAA in combination with 0.05 or 0.10% LAE reduced *L. monocytogenes* by 2.68 ± 0.02 or 2.81 ± 0.01 log_10_ CFU/apple (Figure 2A). However, including Tween 80 or Tween 85, up to 0.20% only slightly improved the efficacy of 80 ppm PAA solution against *L. monocytogenes* on apples, though they were statistically different (*P* < 0.05) (Figure 2B).

Including either LAE, Tween 80, or Tween 85 significantly increased (*P* < 0.05) the wettability of PAA solution on apple surfaces as indicated by decreased contact angles (Figures 2C,D). The PAA solutions with 0.001–0.005% and 0.01% LAE had a contact angle of 76–77° and 68.2°, respectively, which did not differ from those with 0.1 or 0.2% Tween 80/85 (Figures 2C,D). Data indicated that the enhanced anti-*Listeria* efficacy of LAE in PAA solutions might be mainly due to its antimicrobial activity instead of wettability.

### Factors Impact Antimicrobial Strengthening Effects of Lauric Arginate in Peracetic Acid Solutions

**FIGURE 3:**
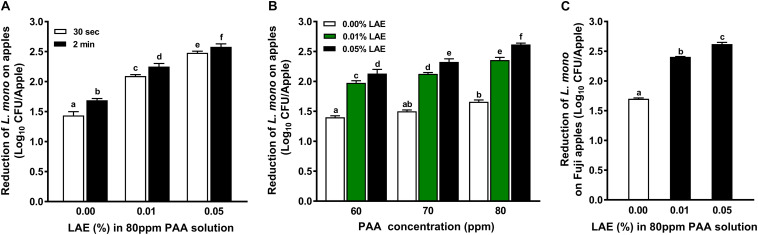
Efficacy of peracetic acid with lauric arginate in reducing *L. monocytogenes* on apples under different concentrations and contact times at 22°C. **(A)** Reduction of *L. monocytogenes* on Granny Smith apples subjected to 80 ppm peracetic acid (PAA) with or without lauric arginate (LAE) for a 30-sec or 2-min contact. **(B)** Reduction of *L. monocytogenes* on Granny Smith apples treated with different PAA and LAE combinations for 2 min. **(C)** Reduction of *L. monocytogenes* on Fuji apples after a 2-min exposure of 80 ppm PAA with different concentrations of LAE. The initial *L. monocytogenes* population on apple surfaces was ∼ 6.8 log_10_ CFU/apple. Mean ± SEM, averaged from three independent experiments; 10 apples per treatment within an independent study. ^a–f^Mean among bars without common letters differ significantly (*P <* 0.05).

Extending the contact time from 30 to 120 sec significantly enhanced (*P* < 0.05) anti-*L. monocytogenes* efficacies of PAA with or without LAE (Figure 3A). Regardless of contact time, the antimicrobial efficacies of PAA solutions against *L. monocytogenes* on GSA increased with elevated LAE concentrations (*P* < 0.05). Increasing LAE concentration to 0.05% caused 2.48–2.58 log_10_ CFU/apple reductions of *L. monocytogenes* on apples (Figure 3A). Decreasing the concentration of PAA from 80 to 60 or 70 ppm diminished (*P* < 0.05) the efficacy of PAA with LAE treatment against *L. monocytogenes* on GSA, regardless of LAE concentration (Figure 3B). The efficacies of PAA with 0.01 and 0.05% LAE against *L. monocytogenes* on Fuji apples were not significantly different from those on GSA; a 2-min exposure to PAA with 0.01–0.05% LAE reduced *L. monocytogenes* on Fuji apples by 2.40–2.62 log_10_ CFU/apple (Figure 3C).

**FIGURE 4:**
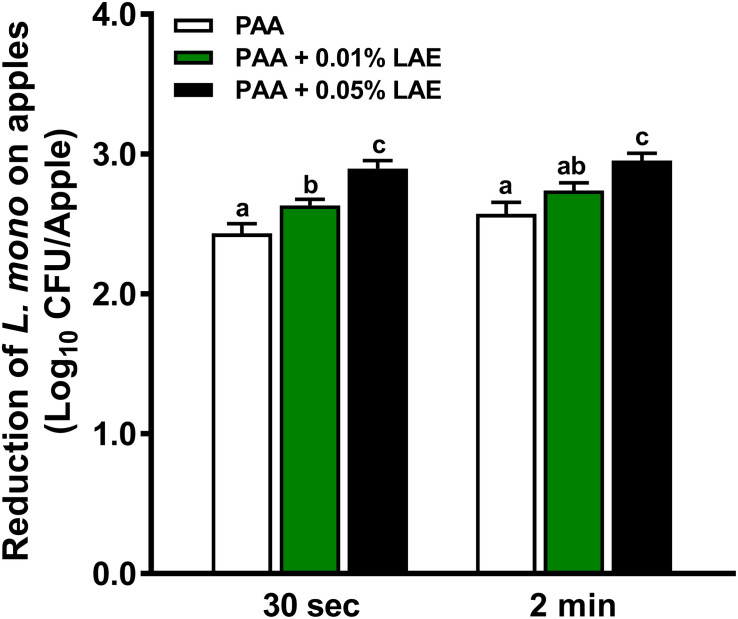
Efficacy of peracetic acid with lauric arginate against *L. monocytogenes* on apples at 46°C. Granny Smith apples subjected to 80 ppm peracetic acid (PAA) alone or in the presence of lauric arginate (LAE) for 30-sec or 2-min contact time. The initial *L. monocytogenes* level on apple surfaces was ∼ 6.5 log_10_ CFU/apple. Mean ± SEM, averaged from three independent experiments; each independent experiment has 10 apples per treatment. ^a–c^Mean among bars without common letters differ significantly (*P <* 0.05).

Peracetic acid with LAE solutions at 46°C showed higher effectiveness against *L. monocytogenes* on GSA than that at 22°C (*P* < 0.05) (Figure 4). A 30–120-sec treatment of PAA at 80 ppm with 0.05% LAE at 46°C reduced *L. monocytogenes* by 2.90–2.95 log_10_ CFU/apple (Figure 4). The contact time has a diminished effect on enhancing effects of LAE in PAA solutions at 46°C compared with that at 22°C (Figures 3A, 4). The strengthening effects of 0.05% LAE on anti-*Listeria* efficacy of PAA, when applied at 46°C, provided an additional 0.54 log_10_ CFU/apple reduction compared with that at 22°C (Figures 3A, 4).

### Verification of Anti-*Listeria* Efficacies of Peracetic Acid With Lauric Arginate Solutions in a Pilot Spray-Bar Brush Bed System

The antimicrobial efficacies of PAA in combination with LAE against *Listeria* on fresh apples were further verified in a pilot spray-bar brush bed system. Spray wash of PAA with 0.01 and 0.05% LAE at RT for 30–120 sec reduced *Listeria* by 1.47–1.64 and 1.68–1.76 log_10_ CFU/apple, respectively (Table 1). Consistently with the lab-scale testing, the addition of LAE significantly improved the antimicrobial efficacy of PAA solution (*P* < 0.05) (Table 1). Increasing the temperature of PAA with 0.01 and 0.05% LAE solution from 22 to 46°C further increased (*P* < 0.05) the efficacies and resulted in 1.83–1.94 and 2.08–2.14 log_10_ CFU/apple reductions of *Listeria* for 30–120 sec contact, respectively (Table 1).

**TABLE 1:** Antimicrobial effectiveness of peracetic acid with lauric arginate solution against *L. innocua* on Granny Smith apples applied in a pilot spray-bar system.

Treatment	Contact time (s)	Reduction (log_10_ CFU/apple)	
		22°C	46°C
PAA	30	0.93 ± 0.05^aA^	1.55 ± 0.05^aB^
	120	1.02 ± 0.04^aA^	1.63 ± 0.06^aB^
PAA + 0.01% LAE	30	1.47 ± 0.06^bA^	1.83 ± 0.03^bB^
	120	1.64 ± 0.05^bcA^	1.94 ± 0.03^bcB^
PAA + 0.05% LAE	30	1.68 ± 0.04^bcA^	2.08 ± 0.04^cB^
	120	1.76 ± 0.05^cA^	2.14 ± 0.04^cB^

*Data are reported as mean ± SEM from three independent replicates. There were 10 apples per treatment within each independent replicate. Mean values within a column without a common letter (superscripted a–c) differ significantly (*P <* 0.05). Mean values within a row without a common letter (superscripted A and B) differ significantly (*P* < 0.05). The initial *L. innocua* population on apple surfaces was ∼ 6.8 log_10_ CFU/apple. *PAA*, peracetic acid (which is used at 80 ppm for all treatments); *LAE*, lauric arginate.*

## Discussion

### Roles of Lauric Arginate in Enhancing Antimicrobial Efficacy of Peracetic Acid Solution Against *Listeria monocytogenes* on Fresh Apples

Lauric arginate has received increasing attention in the fresh produce industry due to its antimicrobial activity against *L. monocytogenes*, used alone or in combination with other antimicrobials. However, the reported effectiveness of LAE against *Listeria* varied among different studies. For example, a 2-min exposure of 0.01% LAE resulted in 3.1 log_10_ CFU/g log reduction of *L. monocytogenes* spot-inoculated on lettuce surfaces with an initial bacterial population of 6.6 log_10_ CFU/g and treated 15-min after inoculation (Nübling et al., 2017). The application of 0.10% LAE for 20 min reduced *L. innocua* by 2.3 log_10_ CFU/cm^2^ on lettuce surfaces, where *L. innocua* was spot inoculated on the lettuce surfaces with an initial bacterial load of 6–8 log_10_ CFU/ml and exposure to LAE treatment 1 h after inoculation (Huang and Nitin, 2017). The antimicrobial strengthening effects of LAE when used with other antimicrobials have been mostly conducted on dairy and meat products (Stopforth et al., 2010; Soni et al., 2012; Ma et al., 2013). The addition of 0.075% LAE with essential oils (cinnamon, eugenol, and thymol oils) caused an additional 2–4 log_10_ CFU/ml reduction of *L. monocytogenes* in 2% reduced-fat milk compared with adding essential oils alone after 4-h treatment (Ma et al., 2013). The addition of 0.07% LAE in a mixture of 1.68% potassium lactate and 0.12% sodium diacetate caused an additional ∼ 1.0 log_10_ CFU/g reduction of *L. monocytogenes* on cooked cured ham after 24-h exposure at 4°C compared with no LAE control (Stopforth et al., 2010). Nonetheless, a recent study on fresh apples reported that the antimicrobial efficacy of 80 ppm PAA with 0.10% LAE against *L. innocua* on apple surfaces was not different from no LAE control (Pietrysiak et al., 2019), which was likely due to large standard deviations among replicates associated with that study. Consistent with studies on dairy and meat products, we herein showed that the addition of LAE significantly enhanced the antimicrobial efficacies of PAA solutions against *L. monocytogenes* in water or on apple surfaces in a concentration-dependent manner. This strengthening effect was further verified in a pilot apple spray-bar brush bed processing line.

The enhanced efficacy of LAE with PAA treatment could be due to their synergic antimicrobial activities. Both PAA and LAE disrupt the lipoprotein cytoplasmic membranes of bacteria and cause the leakage of intercellular components (Maris, 1995; Rodriguez et al., 2004; Becerril et al., 2013). PAA also oxidizes proteins, enzymes, lipids, and DNA by inducing the release of intracellular reactive oxygen species (Leaper, 1984; Maris, 1995; González-Aguilar et al., 2012). Additionally, LAE has an oxidizing property and has been shown to generate oxidative stress against *Escherichia coli* (Yang et al., 2019), which might be synergistic with PAA in releasing bactericidal reactive oxygen species to activate membrane lipid peroxidation and amplify oxidative damage of DNA, ultimately causing cell death. LAE also can interact with bacterial DNA and result in DNA secondary structure changes (Ma et al., 2016).

### Lauric Arginate Wettability in Enhancing the Antimicrobial Efficacy of Peracetic Acid Against *L. monocytogenes* on Apple Surfaces

Lauric arginate, Tween 80, and Tween 85 are surface-active agents that lower the interfacial tension between a liquid and solid surface and increase the spreading property of a liquid solution (Yu et al., 2001; Huang and Nitin, 2017). Consistently, we showed that including LAE, Tween 80, or Tween 85 in PAA solution increased the spreading property on apple surfaces. Of note, the wettability of LAE is stronger than those of Tween 80 or Tween 85; the PAA solutions with 0.10% LAE and 0.10% Tween 80 or Tween 85 reduced contact angle from 99.1 to 41.6, 76.6, or 72.6°, respectively. PAA with 0.10% Tween 80 or Tween 85 solution had a comparable wettability as PAA with 0.005% LAE, but the antimicrobial strengthening effects of 0.005% LAE was much higher than those of PAA with 0.10% Tween 80 or Tween 85, indicating the enhanced anti-*Listeria* efficacies of LAE in PAA solutions should be mainly due to bactericidal reactive oxygen species instead of its wettability (Yang et al., 2019). In support of our findings, LAE is more effective against *L. innocua* on lettuce surface than that of Tween 20 (Huang and Nitin, 2017). Inclusion of 0.10% of Tween 80 in 2.0% citric acid solution failed to increase its antimicrobial efficacy against *Salmonella* on alfalfa seeds (Weissinger and Beuchat, 2000). In contrast, 80 ppm PAA solution with 0.10% Tween 20 and 0.10% LAE had a similar contact angle on Gala apple surface, but 80 ppm PAA with 0.10% Tween 20 exhibited a better antimicrobial efficacy against *L. innocua* on apples than that with 0.10% LAE (Pietrysiak et al., 2019).

### Factors Influence the Antimicrobial Efficacy of Peracetic Acid With Lauric Arginate Solution Against *L. monocytogenes* on Apples

The extended contact time from 30 sec to 2 min significantly increased the efficacy of PAA with LAE solution at RT, but the magnitude of increment was smaller than that of 80 ppm PAA only (Shen et al., 2019). Increased PAA concentration significantly increased the antimicrobial activities of PAA with LAE solutions against *L. monocytogenes* on fresh apples, and the effects of PAA concentration are more pronounced in PAA with LAE solutions compared with PAA alone (Shen et al., 2019). This is possibly due to the increased production of bactericidal reactive oxygen species in PAA with LAE solutions compared with PAA alone.

Our previous study showed that the efficacy of PAA against *L. monocytogenes* on fresh apples was enhanced when it was applied at 46°C, causing an additional 1.02 and 0.89 log_10_ CFU/apple reduction at a 30-sec or 2-min treatment, respectively, compared with those at RT (Shen et al., 2019). The addition of 0.05% LAE in 80 ppm PAA solution at RT obtained a comparable log reduction of *L. monocytogenes* as PAA only solution applied at 46°C (Shen et al., 2019). Similarly, the addition of LAE significantly enhanced PAA anti-*Listeria* efficacy at 46°C, but the strengthening effects were smaller than that applied at RT. The mild heat (Ebrahimi et al., 2018) and LAE (Becerril et al., 2013; Pattanayaiying et al., 2014) caused cellular membrane damages. Thus, the effects of mild heat on the bacterial cell membrane might diminish the strengthening effects of LAE. Spray wash of PAA with LAE in a pilot facility at either 22°C or 46°C was less effective than those applied *via* lab-scale immersion intervention, which is consistent with our previous finding on PAA intervention alone (Zhu et al., 2020). Similar to our findings, LAE combined with eugenol was less effective in reducing *Salmonella* on spinach when conducted by spray wash compared with immersion intervention (Ruengvisesh et al., 2015).

## Conclusion

Low concentrations of LAE significantly enhanced the effectiveness of PAA against *L. monocytogenes* on fresh apples. The antimicrobial efficacies of PAA with LAE treatments increased with the increased concentration of PAA or LAE. The most efficacious treatment was a combination of 80 ppm PAA with 0.05% LAE conducted at 46°C. In this scenario, a 2.90 log_10_ CFU/apple reductions of *L. monocytogenes* on fresh apples was achieved. Data showed that LAE plus PAA is a practical and viable PAA spray-bar brush bed intervention strategy with enhanced anti-*Listeria* efficacy for the apple industry to prevent cross contamination of *L. monocytogenes* of fresh apples, facilitating their compliance with FSMA Preventive Controls requirements.

## Data Availability Statement

The original contributions presented in the study are included in the article, further inquiries can be directed to the corresponding author.

## Author Contributions

XS conducted the experiment, analyzed the data, and wrote the manuscript. JC and JM helped with sample processing. M-JZ and IH revised the manuscript. M-JZ supervised the work, guided the experimental design, analyzed the data, and was in charge of the funding acquisition. All authors contributed to the article and approved the submitted version.

## Conflict of Interest

The authors declare that the research was conducted in the absence of any commercial or financial relationships that could be construed as a potential conflict of interest.
